# Age-Related Differences in Knowledge-Attitude-Practice Patterns of Nonnutritive Sweetener Consumption among Adolescents and Adults: A Cross-Sectional Survey in Southeast Nigeria

**DOI:** 10.1016/j.cdnut.2026.109431

**Published:** 2026-07-02

**Authors:** Beulah F Ortutu, Claire C Okwunodulu, Augusta C Ameze, Joy Sopuru, Adaeze C Ukegbu, Kelechi Ikpemo, Adiah M Odu, Nancy Oriaku, Deborah S Chijioke, Patricia O Ukegbu

**Affiliations:** 1 Department of Nutrition, Texas A&M University, College Station, TX, United States; 2 Department of Human Nutrition and Dietetics, Michael Okpara University of Agriculture, Umudike, Nigeria; 3 Department of Food Science and Technology, Michael Okpara University of Agriculture, Umudike, Nigeria; 4 Department of Medicine, Gregory University, Uturu, Nigeria

**Keywords:** nonnutritive sweeteners, adolescents, knowledge–attitude–practice, nutrition knowledge, information source, ultraprocessed foods, dietary behavior, Nigeria

## Abstract

**Background:**

Nonnutritive sweeteners (NNS) are increasingly incorporated into ultraprocessed foods and beverages, yet psychosocial drivers of NNS consumption across age groups in low- and middle-income settings remain poorly characterized. Understanding how knowledge, attitudinal beliefs, and information sources shape NNS consumption is essential for designing effective nutrition interventions.

**Objectives:**

This study examined age-related patterns of NNS consumption among adolescents and adults and evaluated how knowledge, attitudes, and sources of nutrition information interact to influence consumption practices.

**Methods:**

A cross-sectional study was conducted among 2769 adolescents (10–19 y) and adults (≥20 y) in southeast Nigeria. Data were collected using a structured questionnaire assessing sociodemographic characteristics, NNS-related knowledge, attitudinal beliefs, consumption practices, and information sources. Mediation and conditional process analyses evaluated whether attitudinal beliefs mediated the knowledge-practice relationship and whether this mediation varied by knowledge level, age group, and information source. Logistic regression identified predictors of high NNS consumption.

**Results:**

Adolescents (83.1%) showed a significantly higher prevalence of NNS consumption than adults (16.9%) (*P* < 0.001). Results from the logistic regression revealed that being an adolescent was strongly associated with higher NNS consumption [adjusted odds ratio (AOR) = 19.45; 95% confidence intervals (CI): 15.43, −24.51]. Information sources from health professionals (AOR = 0.67; 95% CI: 0.47, −0.95) and the presence of underlying disease (AOR = 0.50; 95% CI: 0.31, −0.82) were significantly associated with lower consumption. Mediation analysis indicated that positive attitudes partially mediated the relationship between knowledge and consumption practice (indirect effect *P* < 0.05).

**Conclusions:**

NNS consumption patterns exhibited significant age-related variance and are shaped by a complex interplay between knowledge, belief-based attitudes, and the prevailing information environment. Public health nutrition interventions must evolve beyond traditional knowledge dissemination and adopt a multifaceted approach that addresses information quality, food environments, and age-targeted programs.

## Introduction

Global eating practices have changed over the decades [1]. Social, economic, and environmental influences shape eating practices more than individual choice alone. Eating behaviors are increasingly influenced by “who is consuming food,” “when food is consumed,” “where food is consumed,” and “why certain foods are selected” [2]. These influences reflect the combined effects of urbanization, globalization of food systems, and technological innovation in food production [3]. This has driven a transition from traditional diets rich in minimally processed foods toward dietary patterns dominated by energy-dense, refined, and ultraprocessed foods [4]. These changes have contributed to the rising global burden of overweight, obesity, and noncommunicable diseases, which are now among the leading causes of premature mortality worldwide [5,6]. Adolescents are vulnerable within these evolving food environments, as their eating practices are strongly shaped by marketing exposure, peer influence, and limited autonomy over food choices, leading to greater consumption of ultraprocessed foods that increasingly contain nonnutritive sweeteners (NNS) [[7], [8], [9]].

NNS, defined as low- or no-calorie sweeteners that provide little or no energy, including aspartame, sucralose, saccharin, and stevia, are widely used as sugar substitutes in beverages, snacks, confectionery, dairy products, and stimulant drinks [10]. NNS consumption is often habitual, socially reinforced, and embedded within everyday food routines rather than used intentionally as a dietary tool [11]. Although NNS are frequently framed as “healthier” alternatives to sugar, their real-world use is shaped by widespread marketing, unaware substitution, availability, affordability, and taste preferences [12,13]. Among adolescents, NNS-containing products are commonly consumed as part of mainstream food culture, rather than as deliberate substitutes for sugar, raising concerns about how such practices align with broader goals of improving diet quality and reducing dependence on sweet-tasting foods [10,14]. Emerging evidence also suggests that frequent NNS consumption during adolescence may influence appetite regulation, gut microbiota composition, and reward-driven eating behaviors, although findings remain inconsistent [15,16]. In contrast, among adults, NNS consumption is more commonly motivated by weight management or glycemic control [17]. However, excessive or prolonged intake has been associated in some studies with altered glucose metabolism, compensatory energy intake, and metabolic dysregulation in individuals with obesity or insulin resistance [18].

Understanding NNS consumption requires attention to psychosocial drivers of eating behavior, for which the knowledge–attitude–practice (KAP) framework is widely used in nutrition research [19,20]. The KAP framework assumes that knowledge influences attitudes, which in turn shape dietary practices; however, growing evidence suggests that knowledge alone often fails to translate into healthier eating behaviors [21]. Empirical evidence examining age-related differences in knowledge and consumption patterns of NNS remains limited among adolescents and young adults, despite rising exposure to NNS-containing foods and beverages in this population [22]. As a result, insights are often drawn from broader dietary behavior and sugar-reduction studies, which consistently show that younger populations may engage in high consumption of sweetened products despite limited nutrition knowledge [23]. Attitudes toward food products are formed by media messaging [24], social norms [25], and perceived health benefits [26], which may override factual understanding, especially when professional nutrition guidance is limited. This disconnect between knowledge and practice highlights the importance of examining how information sources and attitudes interact to influence eating practices.

Despite the growing presence of NNS in global diets, important gaps remain in the literature. Few studies have examined age-related differences in KAPs related to NNS consumption [22]. Evidence from African populations is limited, even as low- and middle-income countries undergo rapid nutrition transitions marked by increased exposure to ultraprocessed foods [27]. In addition, little is known about how reliance on media-based sources compared with professional sources of nutrition information modify eating practices related to NNS consumption. Using the KAP framework, this study aims to examine age-related differences in knowledge, attitudes, and consumption practices of NNS among adolescents and adults. By identifying age-related differences in these relationships, the study aims to highlight lessons for targeted nutrition education and public health interventions that support healthier eating practices within increasingly complex food environments.

## Methods

### Study design and setting

This cross-sectional study was conducted between January and May 2025 in Abia State, southeast Nigeria. The study area comprises both urban and rural communities and reflects the ongoing nutrition transition characterized by increasing availability and consumption of ultraprocessed foods and NNS. Data were collected within selected secondary schools and universities located across the State.

### Population and sampling

The study population consisted of adolescents and adults affiliated with the 4 selected public secondary schools and the 2 universities, respectively. Adolescents were defined as individuals aged 10 to 19 y who were enrolled as students, while adults were defined as individuals aged 20 y and above who were employed as staff in secondary schools and universities. Participants were eligible for inclusion if they were aged 10 y or older, were present during the data collection period, and provided written informed consent. For participants younger than 18 y, parental consent and participant assent were obtained. Individuals who declined participation or submitted questionnaires with substantial missing data were excluded from the final analysis.

The minimum required sample size was estimated using Cochran’s formula for cross-sectional studies, assuming a 95% confidence level (CI), a margin of error of 5%, and an estimated prevalence of adequate nutrition knowledge of 50% because of the absence of prior local data [28]. To account for potential nonresponse and clustering within schools, the target sample size was increased. A total of 2900 questionnaires were distributed, of which 2769 were completed and included in the final analysis, yielding a response rate of ∼95.5%. Although the sample size calculation was based on prevalence estimation, the large final sample provided sufficient power for multivariable, interaction, and mediation analyses.

A multistage sampling technique was used. In the first stage, 4 secondary schools were purposively selected based on willingness to participate, representation of both urban and rural locations, and inclusion of both junior and senior secondary classes. In the second stage, proportional allocation was used to distribute the sample across schools and class levels according to student population size. In the final stage, students were selected within each class using simple random sampling based on class registers and random number tables. All eligible teaching and nonteaching staff present during data collection were invited to participate and included as adults (>20 y old).

### Ethical considerations

Ethical approval for the study was obtained from the Research and Ethics Committees of the Federal Medical Center, Umuahia, Abia State, for different age groups (FMC/QEH/G.596/Vol.10/833 for adolescents and FMC/QEH/G.596/Vol.10/842 for adults). The study was conducted in full accordance with the ethical principles outlined in the Declaration of Helsinki and Nigeria’s National Code for Health Research Ethics, ensuring respect for participants’ dignity, safety, and autonomy.

Written informed consent was obtained from all adult participants, and parental consent with participant assent was obtained for adolescents. Participation was voluntary, confidentiality was maintained throughout the study, and all data were anonymized before analysis.

### Data collection

Data were collected using a structured, self-administered questionnaire adapted from previously validated KAP instruments related to nutrition and NNS consumption [29]. The questionnaire was modified to reflect locally available NNS-containing foods and beverages and to ensure cultural appropriateness. Before the main survey, the instrument was pilot tested among a subset of participants from a nonspecified school to assess clarity and comprehension. Internal consistency of the KAP scales was evaluated using Cronbach α, with all scales demonstrating acceptable reliability (α ≥0.70) [30].

The questionnaire captured information on sociodemographic characteristics, knowledge of NNS, attitudes toward NNS consumption, reported consumption frequency of NNS-containing foods and beverages, sources of information on NNS, and self-reported health status. Permission to conduct the study was obtained from school administrators before data collection. Eight trained research assistants administered the questionnaires under the supervision of the principal investigator and research team members. Participants completed the questionnaires anonymously during school hours in a classroom or staffroom setting. Participation was voluntary, and confidentiality of responses was assured.

### Variables

Knowledge of NNS was assessed using multiple-choice questions covering 3 domains: identification of NNS, common food and beverage sources, and perceived health effects. Correct responses were defined based on established scientific evidence and product labeling information. For identification items, correct responses referred to accurate recognition of substances classified as NNS (e.g., aspartame, sucralose, saccharin, and stevia). For food and beverage sources, correct responses reflected accurate identification of commonly consumed products known to contain NNS within the study context. For perceived health effects, responses were evaluated against current scientific literature [31]. Responses inconsistent with established evidence were classified as incorrect (Supplemental Table 1).

Each correct response was assigned a score of 1, and incorrect responses a score of 0. Scores were summed to generate a total knowledge score, with higher scores indicating greater knowledge. The total knowledge score ranged from 0 to 8.

For descriptive and mediation analyses, knowledge scores were categorized into poor, average, and good knowledge based on predefined cutoffs. Scores ≤2 were classified as poor knowledge, scores from 3 to 5 as mean knowledge, and scores ≥6 as good knowledge. Good knowledge was used as the reference category to facilitate the interpretation of the direction and magnitude of associations, allowing comparison of lower knowledge levels (average and poor) against the highest knowledge group. Continuous knowledge scores were retained for correlation and regression analyses to preserve statistical power.

Attitudinal beliefs toward NNS were assessed using binary questions capturing participants’ endorsement of NNS substitution, comparative evaluations of NNS compared with sugar reduction, and perceived importance of monitoring NNS content in foods. Responses were summed to generate a composite attitude score, with higher scores reflecting more favorable attitudes toward NNS consumption. Although these items do not constitute a traditional Likert-scale attitude measure, they capture cognitive and normative beliefs related to NNS consumption that are commonly treated as attitudinal components within KAP-based nutrition research [32,33] (Supplemental Table 2).

Practices related to NNS consumption were assessed using a semiquantitative food frequency questionnaire covering 11 commonly consumed NNS-containing food and beverage items identified in the study setting. Participants reported frequency of consumption using predefined categories: never, rarely, monthly, once weekly, 2 to 3 times weekly, >3 times weekly, once daily, 2 to 3 times daily, and >3 times daily (Supplemental Table 3).

To quantify intake, frequency responses were assigned ordinal scores ranging from 1 to 9, with higher values indicating greater consumption frequency (i.e., never = 1, rarely = 2, monthly = 3, once weekly = 4, 2–3 times weekly = 5, >3 times weekly = 6, once daily = 7, 2–3 times daily = 8, >3 times daily = 9). Scores were summed across all 11 items to generate a composite practice score (range: 11–99). For crosstab and regression analysis, the practice score was dichotomized into high consumption (>50) and low consumption (≤50) based on the distribution of the data and to distinguish frequent from less frequent consumers.

The source of information on NNS was assessed by asking participants to identify their primary source from predefined categories: media, health professionals, academic resources, and family and friends. Participants were required to select the most influential or frequently used source. Responses were coded as a categorical variable and used in descriptive, bivariate, and regression analyses.

### Covariates

Age group, sex, residential location, presence of any self-reported disease condition, and primary source of information on NNS were included as covariates based on their potential to confound the relationship between knowledge, attitudes, and NNS consumption. Age group was defined based on participant classification (10–19 y as adolescents; ≥20 y as adults). Residential locations were categorized as urban or rural. Presence of underlying disease was self-reported and coded as a binary variable. The primary source of information was treated as a categorical variable, with family and friends used as the reference category in regression analyses. These variables were selected a priori based on existing literature and their theoretical relevance to dietary behavior and nutrition knowledge.

### Statistical analysis

Data were analyzed using IBM SPSS Statistics (IBM Corporation, version 29). Descriptive statistics were computed for all variables, with continuous variables summarized as means and SDs and categorical variables summarized as frequencies and percentages. Given the large sample size, parametric tests [independent *t*-test and analysis of variance (ANOVA)] were applied, as they are robust to moderate deviations from normality. Spearman rank correlation coefficients were used to examine bivariate associations among KAP scores. Regression-based analyses were conducted to examine associations between KAP scores while accounting for potential confounders. Given the school-based sampling design, Standard Errors (SEs) were applied where appropriate to account for clustering at the school level.

Mediation analyses were performed using the PROCESS macro (version 5) for SPSS to examine whether attitudes toward NNS consumption statistically mediated the association between knowledge and consumption practices [34]. Knowledge was treated as the independent variable, attitude as the mediator, and practice as the outcome variable. Knowledge was treated as a categorical variable with 3 levels: good knowledge (reference group), average knowledge, and poor knowledge. Attitude was coded such that higher scores reflected more positive attitudes toward NNS, and practice was measured as a continuous score. Bootstrapping with 5000 samples was used to estimate indirect effects and corresponding 95% CIs. All mediation analyses were interpreted as statistical associations rather than causal pathways because of the cross-sectional design.

Moderation analyses were conducted using interaction terms within regression models to assess whether associations among KAP differed by age group (adolescents compared with adults), source of information on NNS, and residential location (urban compared with rural).

Logistic regression analysis was used to identify sociodemographic predictors of NNS consumption. Independent variables included age group, sex, place of residence, self-reported disease status, and primary source of information on NNS. Adjusted odds ratios (AORs) and 95% CIs were reported, and multicollinearity among predictors was assessed before model estimation.

Assumptions fundamental to the statistical analyses were assessed before model estimation. Normality of continuous variables was evaluated using visual inspection of histograms and skewness values. Homogeneity of variance for group comparisons was assessed using the Levene test. For regression analyses, linearity and homoscedasticity were examined through residual plots. Multicollinearity among independent variables was assessed using variance inflation factors, with values <10 indicating acceptable levels. For logistic regression models, model fit was evaluated using the Hosmer-Lemeshow goodness-of-fit test. No major violations of assumptions were observed.

## Result

### Demographic characteristics

Table 1 presents the distribution of KAP scores across sociodemographic characteristics. Independent *t*-tests and 1-way ANOVA were used to assess differences in KAP scores between groups to identify potential determinants of NNS-related behaviors. Among the 2769 participants, more than half were females (52.1%), adolescents (58.4%), and resided in the rural areas (66.9%) of the study area. Significant differences in KAP were observed across several sociodemographic characteristics (*P* < 0.05). Adolescents had higher knowledge and practice scores than adults, whereas attitude scores were also significantly higher among younger age groups. Participants residing in urban areas showed higher knowledge and attitude scores, whereas rural participants demonstrated slightly higher practice scores. In addition, individuals who reported family and friends as their primary source of information had higher practice scores compared with those relying on health professionals. The demographic data of all participants are shown in Table 1.

**TABLE 1:** Sociodemographic characteristics of participants and associated knowledge, attitude, and practice scores for NNS

Variable	*N* (%)	Knowledge score		Attitude score		Practice score	
		Mean ± SD	*P* values	Mean ± SD	*P* values	Mean ± SD	*P* values
Gender			0.623		0.050		0.323
Male	1326 (47.9)	3.78 ± 1.59		1.25 ± 1.13		55.48 ± 21.34	
Female	1443 (52.1)	3.75 ± 1.71		1.17 ± 1.04		54.69 ± 20.42	
Age (y)			<0.001∗		<0.001∗		<0.001∗
10–13	668 (24.1)	3.62 ± 1.33		1.37 ± 0.89		65.50 ± 14.07	
14–16	744 (26.9)	4.16 ± 1.28		1.40 ± 1.03		69.52 ± 13.65	
17–25	268 (9.7)	4.18 ± 1.65		1.29 ± 1.15		58.46 ± 16.36	
26–35	291 (10.5)	3.62 ± 1.91		0.97 ± 1.17		46.63 ± 17.64	
36–45	517 (18.7)	3.44 ± 2.03		0.91 ± 1.14		36.75 ± 15.98	
>46	281 (10.1)	3.46 ± 1.85		1.00 ± 1.15		31.18 ± 14.11	
Age group			<0.001∗		<0.001∗		<0.001∗
Adolescents	1617 (58.4)	3.97 ± 1.35		1.39 ± 0.98		66.88 ± 14.12	
Adults	1152 (41.6)	3.48 ± 1.96		0.94 ± 1.15		38.48 ± 17.21	
Occupation			<0.001∗		<0.001∗		<0.001∗
Teaching staff	200 (7.2)	4.89 ± 1.35		1.75 ± 1.13		45.02 ± 16.48	
Nonteaching staff	952 (34.4)	3.18 ± 1.94		0.77 ± 1.08		37.11 ± 17.26	
Student	1617 (58.4)	3.97 ± 1.35		1.40 ± 0.98		66.87 ± 14.12	
Residential location			<0.001∗		<0.001∗		<0.001∗
Rural	1852 (66.9)	3.61 ± 1.70		1.07 ± 1.09		52.99 ± 21.34	
Urban	917 (33.1)	4.08 ± 1.50		1.49 ± 1.00		56.25 ± 19.23	
Presence of any disease condition			0.216		0.065		<0.001∗
Yes	122 (4.4)	3.94 ± 1.60		1.39 ± 1.09		39.66 ± 20.14	
No	2647 (95.6)	3.76 ± 1.65		1.20 ± 1.08		55.77 ± 20.63	
Source of information about NNS			<0.001∗		<0.001∗		<0.001∗
Media (TV, radio, and internet)	830 (30)	4.30 ± 1.42		1.48 ± 0.99		57.54 ± 18.06	
Health professional	942 (34)	3.11 ± 1.91		0.76 ± 1.04		44.53 ± 21.61	
Academic resources	540 (19.5)	4.01 ± 1.20		1.19 ± 1.01		60.93 ± 19.10	
Family and friends	457 (16.5)	3.85 ± 1.45		1.65 ± 1.04		65.36 ± 16.37	
Perception of the general public of the potential risk of NNS			0.017∗		0.557		<0.001∗
Yes	491 (17.7)	3.60 ± 1.83		1.23 ± 1.23		48.84 ± 20.91	
No	2278 (82.3)	3.80 ± 1.61		1.20 ± 1.04		56.41 ± 20.62	

Independent samples *t*-test was used for comparisons between 2 groups, and 1-way analysis of variance was used for comparisons among >2 groups. Statistical significance was set at *P* < 0.05. Given the number of comparisons performed, *P* values are unadjusted and should be interpreted as exploratory. Significant *P* values are indicated with an asterisk.

Abbreviations: NNS, nonnutritive sweeteners; TV, television.

### Food frequency of NNS among adolescents and adults

**FIGURE 1:**
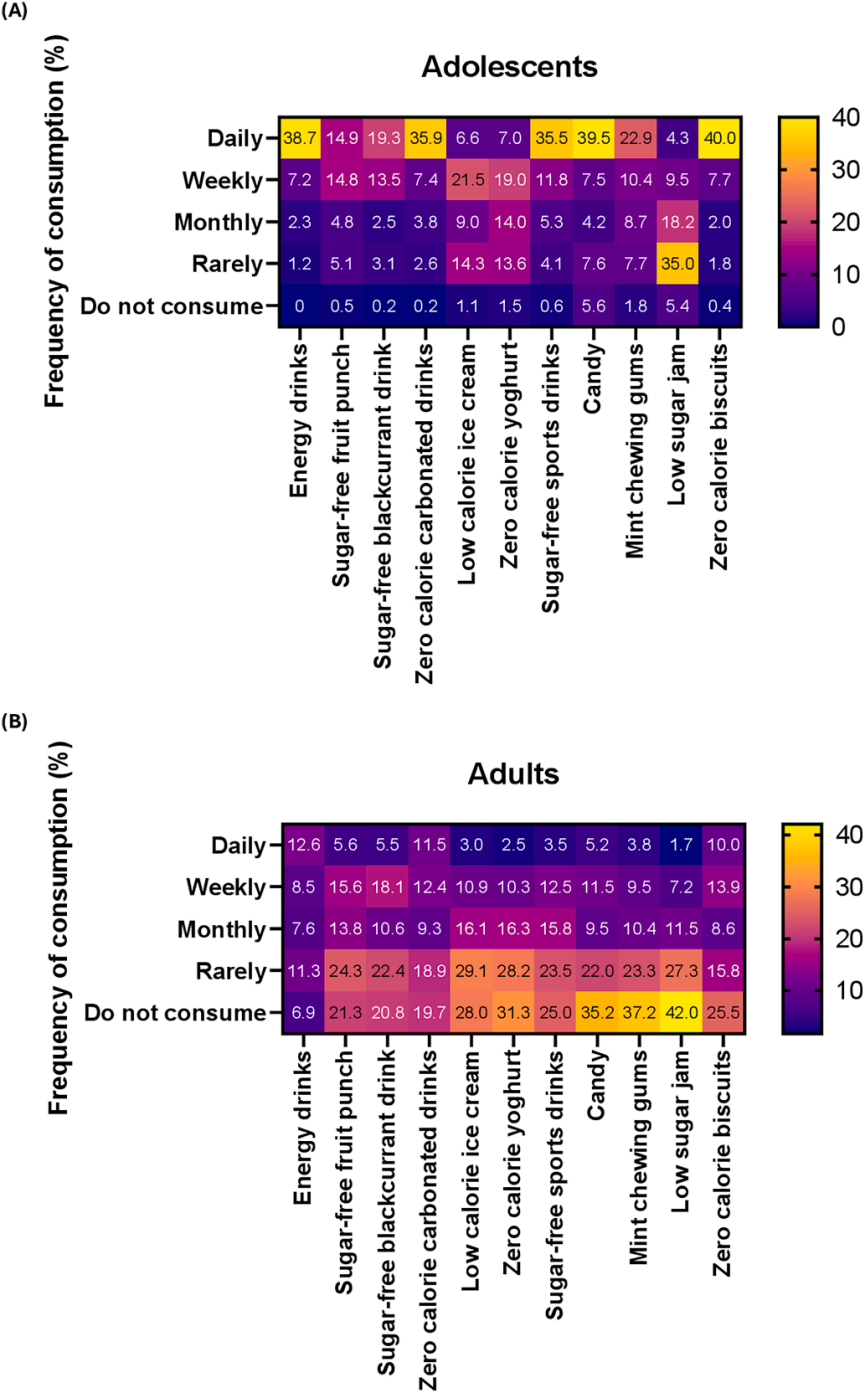
Heatmap showing the frequency of NNS–containing product consumption among (A) adolescents and (B) adults. The heatmap visualizes the proportion of respondents reporting different consumption frequencies (once daily, 2–3 times daily, >3 times daily, weekly, monthly, rarely, or never) for each product category. Darker colors represent higher reported frequencies. Adolescents showed generally higher daily consumption of candies, biscuits, and energy drinks, whereas adults reported predominantly rare or no consumption.

Compared with adults (16.9%), adolescents (83.1%) showed substantially higher NNS consumption (Supplemental Table 4). Among adolescents, the highest daily consumption frequencies were recorded for zero-calorie biscuits (40.0%), candy (39.5%), energy drinks (38.7%), zero-calorie carbonated drinks (35.9%), and sugar-free sports drinks (35.5%). Rare consumption was mainly reported for low-sugar jam (35.0%) (Figure 1A; Supplemental Table 3). In contrast, adults showed generally lower daily consumption across all categories, with 2 to 3 times daily intake of energy drinks (35.7%) being the only NNS with high frequency. Most adults reported rare or no consumption of NNS-containing products (Figure 1B; Supplemental Table 3).

### Relationship between Knowledge, Attitude, and Practice

Spearman correlation revealed a statistically significant positive relationship between knowledge and attitude (ρ = 0.377, *P* < 0.001), attitude and practice (ρ = 0.307, *P* < 0.001), and knowledge and practice (ρ = 0.280, *P* = 0.002) (Table 2). These results support the hypothesized KAP framework and justify the need for a mediation analysis.

**TABLE 2:** Spearman rank correlation coefficients between knowledge, attitude, and practice scores

	Knowledge	Attitude	Practice
Knowledge	1		
Attitude	0.377	1	
Practice	0.280	0.307	1

### Mediation analysis

In Table 3, we examined whether attitude toward NNS consumption mediated the association between knowledge of NNS and practice and whether the effect of attitude on practice was conditional on the level of knowledge. According to Table 3, knowledge of NNS was a significant predictor of attitude toward NNS (*F* = 96.14, *P* < 0.001). The model predicting practice was significant at 50.7% of the variance (*F* = 96.14, *P* < 0.001).

**TABLE 3:** Mediation analysis of the association between knowledge of NNS, attitudes, and consumption practices

	β	SE	95% CI	*P* value
Direct effects				
Average knowledge → attitude	–0.67	0.05	–0.77 to –0.56	<0.001
Poor knowledge → attitude	–1.23	0.06	–1.13 to –1.10	<0.001
Average knowledge → practice	–4.18	0.90	–5.95 to –2.42	<0.001
Poor knowledge → practice	–9.65	1.14	–11.89 to –7.40	<0.001
Conditional effects of attitude				
Good knowledge	0.10	0.63	–1.13 to 1.33	0.872
Average knowledge	2.96	0.35	2.28, 3.64	<0.001
Poor knowledge	2.52	0.75	1.04, 3.99	<0.001
Indirect effects				
Average knowledge → attitude → practice	–1.97	0.27	–2.50 to –1.45	
Poor knowledge → attitude → practice	–3.09	0.83	–4.76 to –1.52	
Total effects (direct and indirect effects)				
Average knowledge → practice	–4.22	0.82	–5.83 to –2.58	
Poor knowledge → practice	–11.10	1.01	–13.10 to –9.13	

Mediation analysis was conducted using regression-based models with knowledge as the independent variable, attitude as the mediator, and practice score as the outcome. Knowledge was entered as a categorical variable (reference: good knowledge). β coefficients represent unstandardized regression estimates. Indirect effects were estimated using bootstrapping with 5000 resamples, and statistical significance was determined based on 95% confidence intervals (CIs) that do not include zero. Conditional effects represent the association between attitude and practice at each level of knowledge. Total effects represent the sum of direct and indirect effects.

Abbreviation: SE: standard error; CI, confidence interval; NNS, nonnutritive sweeteners.

Compared with participants with good knowledge, participants with average knowledge had significantly lower attitude (β = –0.67, 95% CI: –0.77, –0.56, *P* < 0.001) and practice scores (β = –4.18, 95% CI: –5.95, –2.42, *P* < 0.001). Similarly, participants with poor knowledge had lower attitude scores (β = –1.23, 95% CI: –1.35, –1.10, *P* < 0.001) and practice scores (β = –9.65, 95% CI: –11.89, –7.40, *P* < 0.001), suggesting that less knowledge about NNS was associated with more negative attitudes in addition to lower consumption of NNS.

We also assessed the main effect of attitude on practice. No significant effect was observed between the attitude toward NNS and higher consumption of NNS (β = 0.10, 95% CI: –1.13, 1.33, *P* = 0.872), although this model depended on the level of knowledge (*F* = 7.97, *P* < 0.001). Therefore, among participants with average knowledge, a strong positive association between attitude and practice was observed (β = 2.96, 95% CI: 2.28, 3.64, *P* < 0.001). Similarly, among participants with poor knowledge, the effect of attitude on practice was also positive and significant (β = 2.52; 95% CI: 1.04, 3.99; *P* < 0.001). This suggests that attitude toward NNS had little impact on NNS consumption for participants with good knowledge but a stronger influence among those with average or poor knowledge. The mediated effect (*P* < 0.05) of average (β = –1.97, 95% CI: –2.50, –1.45) and poor (β = –3.09, 95% CI: –4.76, –1.52) knowledge through attitude showed a negative impact on practice.

### Influence of socioeconomic factors on the KAP pathway

**FIGURE 2:**
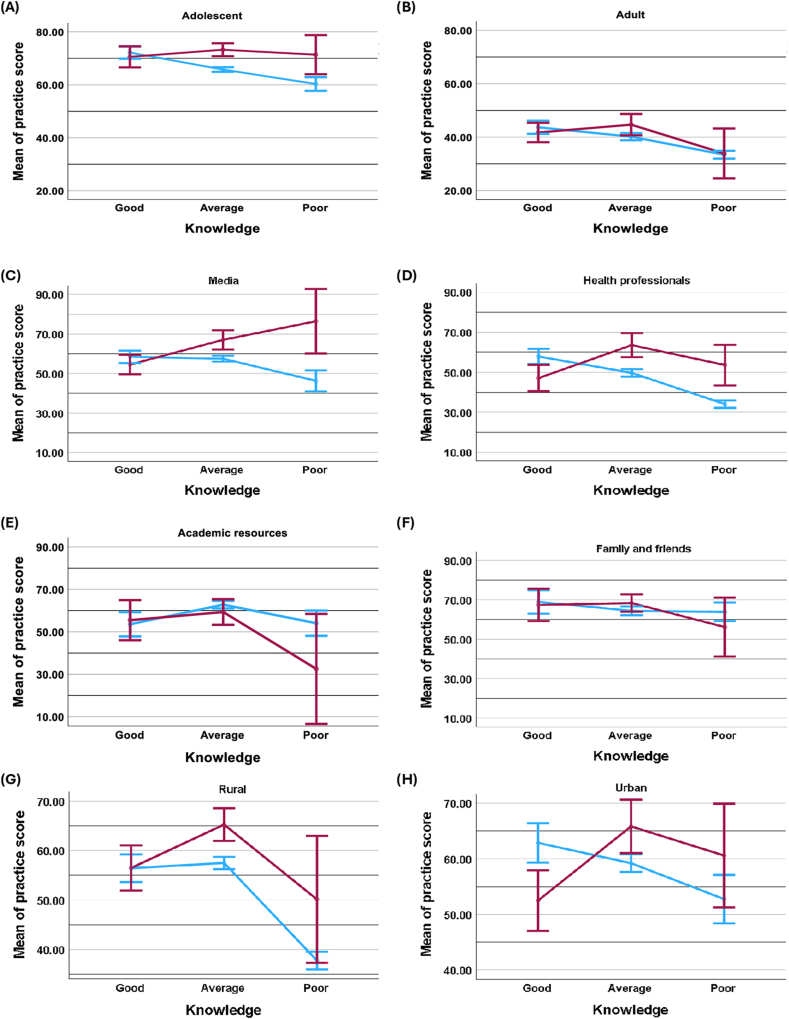
Influence of socioeconomic factors on the knowledge–attitude–practice pathway A 3-way interaction model was used to assess the interaction effect of knowledge, attitude, and socioeconomic characteristics, including age group (A) adolescent (B) adult, getting information from (C) media, (D) health professionals, (E) academic resources, and (F) family and friends, and participants residing in (G) rural and (H) urban areas. Statistical significance was assessed using 3-way analysis of variance (ANOVA), and group differences were identified using Duncan post hoc test. Data are presented as mean ± SE.

Socioeconomic factors shape nutrition behaviors and influence the direction of psychosocial pathways [35]. Therefore, we further examined whether the observed mediation and conditional effects between KAP were modified by participants’ age group, sources of information on NNS, and location. As shown in Figure 2A and 2B, there was a significant 3-way interaction between knowledge, attitude, and age group on practice scores (*F* = 235.23, *P* < 0.001). Among adolescents, participants with poor knowledge showed a positive association between attitude and practice, suggesting that more favorable attitudes were associated with higher NNS consumption. In contrast, adolescents with good knowledge showed little to no change in practice across attitude levels (Figure 2A). Attitude was not significantly associated with practice among adults (Figure 2B). This suggests that adolescents with poor knowledge of NNS are more likely to have positive attitudes to NNS, whereas adults rely more on knowledge than attitude in shaping consumption practices.

We also examined whether the source of NNS knowledge modified the KAP pathway. A significant 3-way interaction was found between knowledge, attitude, and information source (*F* = 7.37, *P* < 0.001). Among participants whose primary source was media (Figure 2C), attitude had a strong positive influence on practice among those with poor or average knowledge, indicating that media exposure can influence attitude when knowledge is limited. For participants who relied on health professionals as their primary source of information (Figure 2D), the relationship between attitude and practice was attenuated, suggesting that knowledge-based guidance may override the influence of attitude. A similar pattern was observed among those who obtained information from academic resources (Figure 2E). In contrast, participants relying on family and friends (Figure 2F) showed weaker and less consistent associations between attitude and practice among those with poor knowledge.

In addition, we assessed whether location modified the relationship between KAP. A significant 3-way interaction was observed between knowledge, attitude, and residential location (*F* = 6.46, *P* < 0.001). Participants with poor or average knowledge showed a strong positive association between attitude and practice, compared with those with good knowledge, among both rural and urban dwellers (Figure 2G–H). Taken together, these interaction effects reveal that the strength of the KAP pathway varies by age group and source of information. Having poor knowledge and a positive attitude increased the consumption of NNS in adolescents and those who got information from the media. These findings indicate that sociodemographic characteristics can shape dietary patterns through knowledge and attitudes.

### Predictors of increased consumption of NNS

Table 4 presents the sociodemographic risk factors for increased NNS consumption, assessed using logistic regression. The findings from the adjusted model suggest that adolescents (AOR: 19.45; 95% CI: 15.43, 24.51) have a 19-fold risk of consuming more NNS than adults. In contrast, those with an underlying disease (AOR: 0.50, 95% CI: 0.31, 0.82) and those who received health information from health professionals (AOR: 0.67, 95% CI: 0.47, 0.95) had a much lower likelihood of consuming NNS.

**TABLE 4:** Adjusted predictors of increased consumption of NNS

Variables	High consumption of NNS			
	OR (95% CI)	*P* value	AOR (95% CI)	*P* values
Gender				
Male	1.10 (0.95, 1.29)	0.208	0.99 (0.81, 1.21)	0.937
Female	—			
Age group				
Adolescents	21.62 (17.71, 26.41)	<0.001	19.45 (15.43, 24.51)	<0.001
Adults	—			
Residential location				
Rural	0.57 (0.48, 0.68)	<0.001	1.11 (0.88, 1.39)	0.370
Urban	—			
Presence of any disease condition				
Yes	0.30 (0.20, 0.44)	<0.001	0.50 (0.31, 0.82)	<0.006
No	—			
Source of information about NNS				
Media (TV, radio, and internet)	0.42 (0.31, 0.56)	<0.001	1.00 (0.71, 1.42)	0.996
Health professional	0.13 (0.10, 0.17)	<0.001	0.67 (0.47, 0.95)	0.026
Academic resources	0.45 (0.33, 0.61)	<0.001	0.69 (0.48, 1.00)	0.051
Family and friends	—			
Perception of the general public’s awareness of the potential risk of NNS				
Yes	0.54 (0.45, 0.66)	<0.001	0.96 (0.74, 1.25)	0.767
No	—			

Adjusted odds ratios (AORs) were adjusted for age group, sex, residential location, presence of disease, and primary source of information in a logistic regression model. Statistical significance was set at *P* < 0.05.

Abbreviations: CI, confidence interval; NNS, nonnutritive sweeteners; OR, odds ratio; TV, television.

## Discussion

This study provides evidence that age shapes the relationship between KAP related to NNS consumption. Using the KAP framework, we show that adolescents and adults differ in the frequency of NNS consumption. These findings highlight that NNS consumption may be influenced by age, information source, and the presence of disease. In our study, adolescents consumed significantly higher amounts of NNS-containing products than adults. This pattern aligns with studies in the United States [36], Portugal [37], South Korea [38], and China [39], reporting increased consumption among younger populations. In contrast, the consumption of NNS among Brazilian adolescents was much lower than that of older adults [40]. Unlike adults, adolescents are unlikely to consume NNS as a conscious strategy for weight management or metabolic health [35]. Instead, consumption appears to be normalized within peer cultures and reinforced through the marketing of stimulant beverages and sweet-tasting products [41].

Although KAPs were positively correlated, our findings reinforce the growing consensus that increased nutrition knowledge does not automatically translate into healthier eating behaviors [21]. Although greater knowledge was associated with more favorable attitudes toward NNS, attitudes did not independently predict consumption among participants with good knowledge. In contrast, attitude had a stronger influence on consumption among adolescents with average or poor knowledge. This suggests that when knowledge is limited, affective responses, such as taste preference, convenience, and brand appeal, may play a dominant role in influencing dietary behavior [42]. Information source was significantly associated with increased consumption of NNS in our study. Participants who relied on media-based sources of nutrition information showed a stronger attitude-practice link, suggesting that exposure to promotional messaging may reinforce positive perceptions of NNS. Conversely, reliance on health professionals or academic sources appeared to affect this relationship, indicating that evidence-based guidance may override media-driven consumption of NNS.

Our study reported higher odds of NNS consumption among adolescents. This may be due to school food environments [43], low-cost availability [10], peer culture [11], and targeted marketing [7] creating conditions that encourage frequent intake of NNS-containing products. The large effect sizes observed in this study show the strength of environmental reinforcement rather than implying direct causality. From a public health perspective, these findings suggest that interventions aimed solely at increasing nutrition knowledge are unlikely to produce meaningful reductions in NNS consumption among adolescents. Effective strategies must address the broader social and environmental determinants of dietary behavior, including food marketing practices, the availability of ultraprocessed products in school settings, and the credibility of nutrition information sources.

This study has several strengths, including a large sample size, age-stratified analyses, and the integration of environmental variables within an analytical framework. However, limitations should be acknowledged. The cross-sectional design prevents causal inference, and self-reported dietary data may be subject to recall or social desirability bias. In addition, the absence of metabolic or clinical outcomes limits conclusions regarding the health consequences of observed consumption patterns. Future longitudinal studies are needed to examine how early-life exposure to NNS influences long-term dietary preferences and metabolic health.

In conclusion, this study reveals that age fundamentally alters how knowledge and attitudes translate into NNS consumption practices. Adolescents with limited knowledge and reliance on media-based information are vulnerable to high consumption. Addressing NNS intake, therefore, requires moving beyond individual knowledge dissemination toward interventions that reshape food environments, regulate youth-targeted marketing, and strengthen access to credible nutrition information. Such approaches are essential for promoting healthier dietary patterns within increasingly complex and commercialized food systems.

## Author contributions

The authors’ responsibilities were as follows; POU: conceptualized, acquired the fund, and supervised the research; BFO, POU: data curation, formal analysis, project administration, data visualization, and methodology; CCO, ACA, JS, AMO, NO, ACU, SDC: data collection; BFO, CCO, POU: original draft writing; BFO, CCO, ACA, JS, AMO, NO, ACU, DDC, POU: reviewed and edited the final draft; and all authors: read and approved the final manuscript.

## Data availability

The datasets used and/or analyzed during the current study are available from the corresponding author on reasonable request.

## Funding

This research was funded by TETFUND and Michael Okpara University of Agriculture, Umudike, Institutional-Based Research (IBR) grant (FNH/03/21) obtained by the principal investigator, Prof. Patricia Ukegbu.

## Declaration of generative AI and AI-assisted technologies in the writing process

The author(s) declare that no generative AI or AI-assisted technologies were used in the writing of this manuscript.

## Conflict of interest

The authors report no conflicts of interest.
