# Supplementary material for: Age-Related Differences in Knowledge-Attitude-Practice Patterns of Nonnutritive Sweetener Consumption among Adolescents and Adults: A Cross-Sectional Survey in Southeast Nigeria
Source: Curr Dev Nutr. 2026 Jul 2;10(8):109431. doi: 10.1016/j.cdnut.2026.109431 (PMC13451885; doi:10.1016/j.cdnut.2026.109431)
Supplement: Supplementary file 1 [file mmc1.pdf]

Age-related Differences in Knowledge-Attitude-Practice patterns of Non-Nutritive sweetener Consumption among adolescents and adults: A Cross-Sectional Survey in Southeast Nigeria.

Beulah F. Ortutu

Supplementary Table 1. Knowledge and perceived health effects of NNS

Variable	Frequency	Percentage (%)
**Awareness of different types of NNS available (e.g. aspartame, stevia, saccharin, sucralose)**		
Yes[a]	1163	42
No	1606	58
**Knowledge of common foods and beverages that contain NNS**		
Yes[a]	1990	71.9
No	779	28.1
**NNS contain fewer calories than sugar**		
Yes[a]	1061	38.3
No	1708	61.7
**Our body senses NNS the same as natural sugar**		
Yes	1216	43.9
No[a]	1553	56.1
**NNS may be associated with a lower risk of cancer**		
Yes	779	28.1
No[a]	1990	71.9
**NNS leads to decreased craving for food**		
Yes	1075	38.8
No[a]	1694	61.2
**NNS helps to decrease tooth decay**		
Yes	720	26
No[a]	2049	74
**NNS helps reduce weight**		
Yes[a]	727	26.3
No	2042	73.7

[a] Scientifically accurate response used to compute knowledge scores; NNS: Non-Nutritive sweeteners

Supplementary Table 2. Attitude towards health risks associated with the consumption of NNS

Variable	Frequency	Percentage (%)
**Daily use of sugar should be substituted with NNS**		
Yes	858	29.9
No	1941	70.1
**Carbonated drinks should be substituted with "diet" drinks**		
Yes	1312	47.4
No	1457	52.6
**NNS is a better option than the absolute removal of all sugars from the diet**		
Yes	473	17.1
No	2296	82.9
**It is important to check whether food labels contain NNS**		
Yes	2089	75.4
No	680	24.6

NNS: Non-Nutritive sweeteners

Supplementary Table 3. Frequency distribution of NNS consumption by age group

Variable	Adolescents		Adults	
	Frequency	Percentage (%)	Frequency	Percentage (%)
**Energy drinks**				
Once daily	625	38.7	145	12.6
2 – 3 times daily	258	16	411	35.7
>3 times daily	142	8.8	10	0.9
Once weekly	116	7.2	98	8.5
2 – 3 times weekly	132	8.2	127	11
>3 times weekly	288	17.8	64	5.6
Monthly	37	2.3	87	7.6
Rarely	19	1.2	130	11.3
Do not consume	0	0	80	6.9
**Sugar-free fruit pouch**				
Once daily	241	14.9	65	5.6
2 – 3 times daily	236	14.6	16	1.4
>3 times daily	112	6.9	14	1.2
Once weekly	239	14.8	180	15.6
2 – 3 times weekly	193	11.9	131	11.4

>3 times weekly	429	26.5	62	5.4
Monthly	77	4.8	159	13.8
Rarely	82	5.1	280	24.3
Do not consume	8	0.5	245	21.3
**Sugar-free blackcurrant drink**				
Once daily	312	19.3	63	5.5
2 – 3 times daily	238	14.7	19	1.6
>3 times daily	70	4.3	9	0.8
Once weekly	218	13.5	208	18.1
2 – 3 times weekly	217	13.4	153	13.3
>3 times weekly	468	28.9	80	6.9
Monthly	41	2.5	122	10.6
Rarely	50	3.1	258	22.4
Do not consume	3	0.2	240	20.8
**Zero-calorie carbonated drinks**				
Once daily	581	35.9	133	11.5
2 – 3 times daily	281	17.4	27	2.3
>3 times daily	154	9.5	22	1.9
Once weekly	120	7.4	143	12.4
2 – 3 times weekly	153	9.5	193	16.8
>3 times weekly	222	13.7	82	7.1
Monthly	61	3.8	107	9.3
Rarely	42	2.6	218	18.9
Do not consume	3	0.2	227	19.7
**Low-calorie ice cream**				
Once daily	106	6.6	35	3
2 – 3 times daily	151	9.3	10	0.9
>3 times daily	150	9.3	7	0.6
Once weekly	348	21.5	126	10.9
2 – 3 times weekly	256	15.8	83	7.2
>3 times weekly	213	13.2	49	4.3
Monthly	145	9	185	16.1
Rarely	231	14.3	335	29.1
Do not consume	17	1.1	322	28
**Zero-calorie yoghurt**				
Once daily	113	7	29	2.5
2 – 3 times daily	155	9.6	17	1.5
>3 times daily	112	6.9	9	0.8
Once weekly	307	19	119	10.3

2 – 3 times weekly	236	14.6	60	5.2
>3 times weekly	223	13.8	44	3.8
Monthly	226	14	188	16.3
Rarely	220	13.6	325	28.2
Do not consume	25	1.5	361	31.3
**Sugar-free sports drinks**				
Once daily	574	35.5	40	3.5
2 – 3 times daily	257	15.9	26	2.3
>3 times daily	178	11	16	1.4
Once weekly	191	11.8	144	12.5
2 – 3 times weekly	131	8.1	126	10.9
>3 times weekly	126	7.8	59	5.1
Monthly	85	5.3	182	15.8
Rarely	66	4.1	271	23.5
Do not consume	9	0.6	288	25
**Candy**				
Once daily	638	39.5	60	5.2
2 – 3 times daily	219	13.5	22	1.9
>3 times daily	117	7.2	12	1
Once weekly	122	7.5	132	11.5
2 – 3 times weekly	109	6.7	95	8.2
>3 times weekly	130	8	64	5.6
Monthly	68	4.2	109	9.5
Rarely	123	7.6	253	22
Do not consume	91	5.6	405	35.2
**Mint chewing gums**				
Once daily	371	22.9	44	3.8
2 – 3 times daily	260	16.1	15	1.3
>3 times daily	160	9.9	19	1.6
Once weekly	168	10.4	110	9.5
2 – 3 times weekly	176	10.9	90	7.8
>3 times weekly	188	11.6	58	5
Monthly	141	8.7	120	10.4
Rarely	124	7.7	268	23.3
Do not consume	29	1.8	428	37.2
**Low sugar jam**				
Once daily	69	4.3	20	1.7
2 – 3 times daily	95	5.9	12	1
>3 times daily	51	3.2	10	0.9

Once weekly	153	9.5	83	7.2
2 – 3 times weekly	126	7.8	59	5.1
>3 times weekly	175	10.8	36	3.1
Monthly	295	18.2	133	11.5
Rarely	566	35	315	27.3
Do not consume	87	5.4	484	42
**Zero-calorie biscuit**				
Once daily	646	40	115	10
2 – 3 times daily	377	23.3	32	2.8
>3 times daily	300	18.6	32	2.8
Once weekly	124	7.7	160	13.9
2 – 3 times weekly	70	4.3	153	13.3
>3 times weekly	33	2	85	7.4
Monthly	32	2	99	8.6
Rarely	29	1.8	182	15.8
Do not consume	6	0.4	294	25.5

NNS: Non-Nutritive sweeteners

Supplementary Table 4. Prevalence of NNS consumption

	**High practice score (>50) F (%)**	**Low Practice score (<50) F (%)**	**Total F (%)**	**p-value**
**Age group**				<0.001*
Adolescents	1420 (83.1)	197 (18.6)	1617 (58.4)	
Adults	288 (16.9)	864 (81.4)	1152 (41.6)	

*Asterisks represent statistically significant associations P < 0.05. Fisher's exact test was used to assess significant associations; NNS: Non-Nutritive sweeteners; F (%): Frequency (percentages)
